# Analysis of Ruck Contact Exposure and Technical–Tactical Behaviors Related to Match Outcome in International Rugby Union

**DOI:** 10.3390/sports14080333

**Published:** 2026-08-03

**Authors:** Diego Hernán Villarejo-García, Carlos Navarro-Martínez, Pedro Lopez Baro, Gabriel Daza-Sobrino, Sebastian Del Rosso, José Pino-Ortega

**Affiliations:** 1 Faculty of Sport Science, University of Murcia, 30100 Murcia, Spain; dvillarejo@um.es (D.H.V.-G.); pedro.lopezb@um.es (P.L.B.); josepinoortega@um.es (J.P.-O.); 2 Education, Diversity and Quality Research Group, Faculty of Education, University of Murcia, Campus de Espinardo, 30100 Murcia, Spain; 3 National Institute of Physical Education of Catalonia (INEFC), University of Barcelona (UB), 08038 Barcelona, Spain; gdaza@gencat.cat; 4 Research Center in Clinical Biochemistry and Immunology (CBICI-CONICET), National Scientific and Technical Research Council, National University of Córdoba, Córdoba 5000, Argentina; sdelrosso@mi.unc.edu.ar

**Keywords:** performance analysis, logistic regression, breakdown, match outcome, rugby union

## Abstract

This study examined the association between technical–tactical ruck variables and the probability of defeat in international rugby union. Six variables (Pitch Territory, Number of Phases, Match Half, Player Type, Gainline Progression/Ruck Position, and Number of Players in the Ruck) were recorded from 2575 rucks across 26 international matches played by the Spanish national team. The binary logistic regression model displayed a significant overall fit (χ^2^ = 137.247, *df* = 9, *p* < 0.001, Nagelkerke *R*^2^ = 0.0692, AUC = 0.6208), achieving 57.98% global accuracy. A high Number of Phases (OR = 3.265 for ≥7 Phases), playing within Own Territory (OR = 1.481), operating Behind Gainline (OR = 1.478), or On Gainline (OR = 1.369), and actions during the Second Half (65.7%) compared to the First Half (54.5%) were significantly associated with higher odds of defeat. Conversely, the post hoc GLMM sensitivity check showed that the associations for these variables remained broadly consistent, while indicating that the exclusive involvement of Backs was associated with the highest estimated probability of defeat among the Player Type categories (64.4% vs. 57.2% for Forwards), and that the involvement of ≥4 players in the ruck became statistically significant in the mixed-effects model after accounting for match-level clustering and was associated with a higher estimated probability of defeat. In conclusion, Number of Phases, Pitch Territory, Gainline Progression, and the quantity and Player Type involved are significantly associated with defeat, aligning with specific contact exposure environments.

## 1. Introduction

In epidemiological studies, it has been evidenced that contact and collisions are the primary mechanisms of injuries in Rugby Union [1,2]. In this sense, the ruck is a game phase characterized by a high frequency of collisions per minute, which produces constant contact exposure [3]. Within elite rugby union, grouped play situations such as the ruck represent highly demanding contact scenarios, consistently identified by literature as critical phases for collision accumulation. Because players frequently engage in physical contests during the breakdown to secure or retain ball possession, the ruck inherently functions as a primary open-play contact exposure environment. Consequently, analyzing the technical–tactical factors that modulate player behaviors during this phase is relevant not only for performance optimization but also for understanding the structural contexts under which contact exposure occurs.

Concurrently, from a performance perspective, the ruck has been considered a decisive action [4,5,6], given that it is one of the game phases in which ball possession is contested [7]. For instance, losing teams tend to generate a higher number of rucks [8,9,10,11], longer ruck durations, and a greater number of players compared to winning teams [6,12,13]. These findings suggest that losing teams might face greater difficulty in overcoming the opponent’s defensive line, resulting in longer possessions and the involvement of a higher number of players [13,14].

Based on the aforementioned evidence, it can be hypothesized that if losing teams are characterized by executing more rucks, more phases, and accumulating more players, they may be exposed to physical collisions longer than winning teams [15]. Therefore, the tactical difficulties that losing teams experience during this game phase represent not only a strategic problem but can also be translated into a pattern that multiplies contact and collision exposure situations, which literature frequently links to potential physical exhaustion and injury outcomes.

Despite the growing interest in performance and injury studies in rugby, investigations directly linking the final match outcome with contact exposure conditions during the ruck remain limited. Consequently, it remains poorly understood how specific ruck variables are associated with the probability of winning or losing a match in high-level competition. To address this gap, the use of a pooled logistic regression framework can help clarify the probabilistic relationship between ruck behaviors and match outcomes (victory or defeat) in rugby matches [16,17,18]. Thus, exploratory classification models can be established to enhance situational match analysis [16].

To establish a robust analytical framework, the selection of breakdown descriptors must be explicitly justified based on their tactical and operational relevance. Contextual and temporal variables, such as Pitch Territory and Match Half, may capture contextual patterns potentially related to accumulated physical fatigue and changes in defensive pressure throughout the match. The Number of Game Phases serves as a direct measure of possession duration and offensive efficiency. Concurrently, operational variables describing the internal structure of the breakdown, specifically Player Type, Gainline Progression, and Number of Players in the Ruck, provide a precise description of spatial optimization, ball recycling speed, and positional vulnerability during the contact contest. Separating these dimensions allows for a comprehensive assessment of how specific strategic choices correlate with final outcomes.

Despite the extensive body of literature focused on rugby union performance analysis, a distinct research gap remains regarding how final match results are associated with these specific technical–tactical and contact exposure variables during open-play breakdowns in senior international rugby competitions. Longitudinal data exploring these specific micro-behaviors within emerging international contexts remain highly constrained.

To address this gap in the scientific literature, the following specific research question was formulated: To what extent are localized ruck contact exposure characteristics and operational tactical behaviors associated with the probability of match defeat in high-level international rugby union matches?

Consequently, the primary objective of this study was to examine the relationship between selected technical–tactical ruck variables (Pitch Territory, Number of Phases, Match Half, Player Type, Gainline Progression, and Number of Players in the Ruck) and match outcome (victory or defeat) in an international senior men’s team across four consecutive competitive seasons.

Based on contemporary performance analysis trends, it was tentatively hypothesized that ruck variables reflecting offensive stagnation or spatial inefficiency, specifically prolonged phase sequences (≥7 phases), failure to clear the gainline, or overpopulating the breakdown (≥4 players), would display a significant statistical association with an increased probability of match defeat, indicating that unrewarded contact exposure tracks alongside adverse competitive outcomes.

## 2. Materials and Methods

### 2.1. Design

This study was retrospective, descriptive, and correlational [19]. The study followed the STROBE guidelines (see Supplementary Material). The performance of a team in competition was analyzed, allowing the identification of the relationship between variables and the match outcome without manipulation of the conditions [14]. A point, idiographic, and multidimensional (P/I/M) observational design with an intra-sessional approach was used. Although the analysis covered different competitive seasons, no longitudinal monitoring of the participants was implemented. The point-based nature of the design was based on the analysis of matches played by a single team during the Rugby Europe Championship; the idiographic dimension was sustained by treating all participants as a cohesive unit of analysis; finally, the multidimensional nature was manifested in the evaluation of different levels of behavioral response [20].

### 2.2. Matches and Technical Actions Analysed

A total of 2575 rucks were analyzed, distributed across 26 matches (10 lost and 16 won) played by the Spanish men’s senior national team in the Rugby Europe Championship during the 2022, 2023, 2024, and 2025 editions. The unit of analysis was the ruck, defined according to Law 15 of the World Rugby regulations [21] as “a phase of play where one or more players from each team, who are on their feet, in physical contact, close to the ball on the ground.”

Since the study was based on data observed and recorded through publicly broadcast match footage, individual informed consent from players or teams was not required [22]. Data access and processing were conducted strictly for academic research purposes and complied with current regulations for the use of public sports data [23].

The inclusion criteria for the analyzed matches were: the inclusion of the matches within the official Rugby Europe Championship tournament; the complete availability of the audiovisual material corresponding to each match; and a sufficient image quality to guarantee the viability of a full analysis.

### 2.3. Materials and Instruments

For data collection, a selection of variables defined in the observation instrument designed and validated by Villarejo et al. [24] was used. The variables considered were: *Pitch Territory:* The zone of the playing field where the ruck took place, categorized as the half of the pitch defended by the team (Own Territory) or the half of the pitch attacked by the team (Opponent Territory). Number of Phases: Consecutive play sequences performed by the attacking team maintaining ball possession between two stoppage points of open play. The categories distinguished between 1–3 Phases, 4–6 Phases, and ≥7 Phases. Match Half: Each of the two regulation halves of the match [21], differentiating between First Half and Second Half. Player Type: The playing position of the player(s) participating in the ruck, categorized into Forwards, Backs, and Backs & Forwards. Gainline Progression/Ruck Position: The location of the ruck in relation to the gainline (the imaginary line of advance that the attacking team needed to overcome). The categories included: Behind Gainline, On Gainline, and Gainline Cleared (In Front). Number of Players in the Ruck: The total number of players from the team in possession of the ball who met the regulatory definition of a ruck. The categories were 1–3 Players and ≥4 Players.

### 2.4. Procedure

In this research, video recordings registered by Radio Televisión Española (available at https://www.rtve.es/play/videos/rugby/ (accessed on 2 June 2026) from the 2022, 2023, 2024, and 2025 seasons of the Spanish men’s senior national team in the Rugby Europe Championship tournament were used. The matches were viewed on screen, and data logging was executed utilizing the Lince observational software (Version 1.4) [22], following the methodological framework proposed by Villarejo et al. [25]. Three observers, each holding a Level 3 coaching certification from the International Rugby Board and possessing 10 years of experience in rugby coaching and match analysis, carried out the observational analyses. Prior to the match observation phase, the observers underwent a rigorous training protocol with the observation instrument, adhering to the guidelines outlined by Losada and Manolov [26]. To guarantee the scientific reliability of the observational data, a rigorous double-blind training and calibration protocol was executed in accordance with the guidelines outlined by Losada and Manolov [26]. Following the initial training phase, a random subset comprising 258 rucks (10.02% of the total final sample) was extracted and analyzed by the observers to compute formal data quality statistics. Inter-observer reliability assessments using Cohen’s Kappa (*k*) yielded highly satisfactory individual values across all six modeled factors: Pitch Territory (*k* = 0.88), Number of Phases (*k* = 0.85), Match Half (*k* = 1.00), Player Type (*k* = 0.82), Gainline Progression (*k* = 0.84), and Number of Players in the Ruck (*k* = 0.89). Concurrently, intra-observer reliability assessed fourteen days later demonstrated strong internal consistency, with individual variable Kappa values ranging from 0.91 to 0.97. The observers performed their primary analyses independently, systematically logging the technical–tactical actions into a validated data matrix, with listwise deletion subsequently applied to ensure data completeness.

### 2.5. Data Analysis

A multiple binary logistic regression analysis was conducted [27] to assess the relationship between the variables observed in the ruck and the match outcome (winner/loser). No a priori sample size calculation was executed due to the retrospective and observational nature of the competitive dataset. However, the exact mathematical adequacy of the sample size for multivariable logistic regression modeling was thoroughly verified using the Events Per Variable (EPV) ratio. Based on our final data matrix, a total of 1334 ruck observations corresponded to defeated matches (51.8% of the total sample). With nine estimated regression coefficients corresponding to the six technical–tactical predictor variables, the Events Per Variable (EPV) ratio at the observational level was 148.22 (1334/9), exceeding the standard threshold of 10 recommended in multivariable modeling literature [28,29]. However, while this reflects a substantial overall volume of ruck-level observations, the amount of independent outcome information is inherently constrained by the total number of matches (*n =* 26) and the nested structure of the data, a hierarchy that was addressed through the post hoc mixed-effects model. The dependent variable was match outcome, coded as 1 for the losing team and 0 for the winning team. The predictor variables included field territory, number of phases, match half, player position, ruck position, and number of players involved in the ruck. The multiple binary logistic regression model was formulated as follows:

$$\ln\left(\frac{p}{1-p}\right)=\beta_{0}+\beta_{1}X_{1}+\beta_{2}X_{2}+\cdots +\beta_{6}X_{6}$$

where *p* represented the probability of the team being classified as the losing team.

The global model fit was verified using the omnibus sequential likelihood ratio test, showing a highly significant overall fit (*χ*^2^ = 137.247, *df* = 9, *p* < 0.001; Nagelkerke *R*^2^ = 0.0692; deviance = 3212.4). Multicollinearity among predictors was checked using the Variance Inflation Factor (VIF); all values remained below 1.15 (ranging from 1.01 to 1.14), ensuring the absence of significant collinearity violations. The model’s classification performance was assessed using a standard cutting point of 0.5, yielding an overall classification accuracy of 57.98% (with class-specific accuracy of 56.00% for victories and 59.82% for defeats). Under defeat coded as the primary event (1), sensitivity was 0.598 and specificity was 0.560. Finally, the discriminative power of the model was assessed using the Receiver Operating Characteristic (ROC) curve metrics, which revealed an Area Under the Curve (AUC) of 0.6208. Additionally, to account for the potential dependency of rucks nested within the same match, a post hoc sensitivity analysis was performed by fitting a Generalized Linear Mixed Model (GLMM) with Match ID (Partido) entered as a random intercept component. The GLMM incorporated the same fixed technical–tactical predictors as the primary model, allowing for a side-by-side comparison of standard errors and adjusted Odds Ratios to assess whether the principal associations remained broadly consistent after accounting for match-level clustering. Random effect distribution was quantified via the random intercept variance (τ_00_) and the Intraclass Correlation Coefficient (ICC). All statistical computing procedures, unpooled logistic regression, and mixed-model estimation algorithms were executed utilizing R software (Version 4.4.0) via the lme4, car, and pROC packages.

## 3. Results

A total of 2575 rucks were analyzed, with 1241 (48.2%) occurring in matches resulting in a victory and 1334 (51.8%) in a defeat. Descriptively, the distribution of breakdown events showed logical strategic variations across all independent operational criteria. Regarding territorial distribution, a total of 1153 rucks (44.8%) were executed within Own Territory, while 1422 (55.2%) took place in Opponent Territory. Temporally, the breakdown volume was heavily concentrated in the introductory phases of play, with 1757 rucks (68.2%) contested during the First Half compared to 818 (31.8%) during the Second Half. In terms of sequence complexity, short series of 1–3 Phases accounted for the majority of the sample with 1601 events (62.2%), followed by 4–6 Phases with 793 rucks (30.8%), and prolonged sequences of ≥7 Phases with 181 events (7.0%). Organizationally, mixed structural interventions involving both Backs and Forwards dominated the tactical environment with 1460 rucks (56.7%), followed by sequences restricted to Forwards 673 rucks (26.1%) and Backs 442 rucks (17.2%). Tactically, local gainline progression indicators revealed that 1250 breakdowns (48.5%) occurred when the gainline was cleanly broken, while 807 (31.4%) were contested On the Gainline, and 518 (20.1%) took place. Finally, regarding defensive and offensive density metrics, maintaining a low occupancy of 1–3 Players in the ruck was the most prevalent behavior with 2192 events (85.1%), contrasted with 383 rucks (14.9%) where the breakdown was overpopulated by ≥4 Players.

Table 1 shows the regression coefficients (*β*), standard errors, *p*-values, odds ratios (OR), and 95% confidence intervals (CI) yielded by both the standard Logistic Model and the post hoc multilevel GLMM framework.

**Figure 1:**
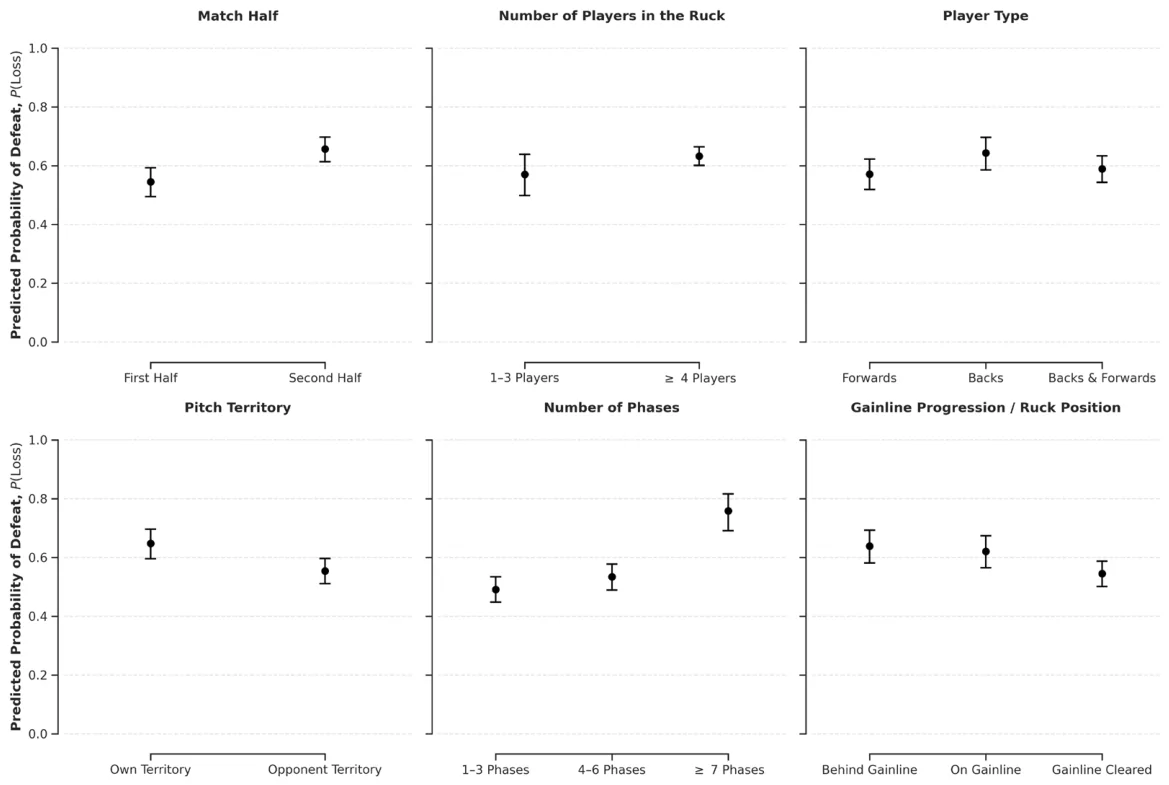
Estimated marginal means plots representing the contextual probabilities of match defeat (P(Loss)) on a scale of 0–1 across the analyzed technical–tactical factors. Whiskers represent the calculated 95% Wald confidence intervals.

The adjusted Odds Ratios (OR) presented in Table 1 represent the estimated change in the odds of the team suffering a match defeat for each specific category shift within the predictors, holding all other model covariates constant. To facilitate direct practical interpretation and avoid confusing population frequencies with model parameters, the logit-scale regression estimates were mathematically transformed into absolute predicted probabilities (P(Loss)) via the extraction of Estimated Marginal Means. This analysis revealed that undergoing a prolonged sequence of ≥7 Phases exhibited the highest absolute probability of ending in match defeat (75.9%; 95% CI: 69.1%, 81.6%) compared to short sequences of 1–3 Phases (49.1%; 95% CI: 44.8%, 53.4%) and intermediate sequences of 4–6 Phases (53.4%; 95% CI: 48.9%, 57.8%). Regarding territorial constraints, executing rucks within Own Territory was associated with an absolute predicted defeat probability of 64.8% (95% CI: 59.6%, 69.7%), whereas breakdowns in Opponent Territory displayed a baseline probability of 55.4% (95% CI: 51.1%, 59.7%). Positionally, breakdowns restricted to Forwards showed a predicted probability of match loss of 57.2% (95% CI: 51.9%, 62.3%), which increased significantly to 64.4% (95% CI: 58.6%, 69.7%) during sequences involving Backs, while mixed structures (Backs & Forwards) yielded a probability of 59.0% (95% CI: 54.4%, 63.4%). Tactically, rucks formed Behind the Gainline and On the Gainline yielded localized defeat probabilities of 63.9% (95% CI: 58.1%, 69.3%) and 62.1% (95% CI: 56.5%, 67.4%) respectively, contrasted with Gainline Cleared scenarios (54.5%; 95% CI: 50.1%, 58.8%). Temporally, rucks contested during the First Half displayed an absolute loss probability of 54.5% (95% CI: 49.5%, 59.3%), whereas a higher baseline of 65.7% (95% CI: 61.4%, 69.8%) was observed during the Second Half. Finally, overpopulating the breakdown with ≥4 Players resulted in an adverse probability of 63.3% (95% CI: 60.1%, 66.4%), while maintaining a low density of 1–3 Players showed a predicted probability of 57.1% (95% CI: 49.9%, 63.9%). These absolute probability profiles remained robust and consistent when adjusted within the post hoc GLMM framework to account for match-level random variance. These absolute probability distributions and their respective 95% confidence intervals are visually projected across the individual panels of Figure 1 to allow for a comprehensive, standalone interpretation of the breakdown environment.

## 4. Discussion

The purpose of this study was to determine the association between ruck variables and the occurrence of defeat in an international rugby union team during the Rugby Europe Championship. To achieve this, a pooled logistic regression analysis was performed alongside a post hoc multilevel Generalized Linear Mixed Model (GLMM) sensitivity check to control for the nested dependency of rucks within individual matches. The findings suggest that these technical–tactical variables display a statistically significant association with the match outcome.

The developed full-sample primary model displayed a global classification capacity characterized by an AUC of 0.6208, a Nagelkerke *R*_2_ of 0.0692, and an omnibus fit of *χ*^2^ = 137.247 (*df* = 9, *p* < 0.001), consistent with the multifactorial nature of match outcomes in team sports [17,30,31,32,33]. While the global classification accuracy was 57.98%, the distribution between victories (48.2%) and defeats (51.8%) across the classification matrix indicates that the model reflects variations in tactical patterns associated with losing outcomes. The post hoc GLMM revealed a match-level random intercept variance of τ_00_ = 0.9033 (*SD* = 0.9504) and an Intraclass Correlation Coefficient (ICC) of 0.2154. Rather than attributing the remaining variance exclusively to the measured variables, this ICC value indicates a meaningful within-match dependency. This statistically supports the necessity of accounting for nested data structures via random factors to confirm the structural stability of the analyzed population-averaged predictors, while cautiously acknowledging the presence of unobserved match-play heterogeneity and unmeasured tactical variations.

Sustained by this controlled analytical baseline, one of the main findings highlights the association between the Number of Phases and the probability of defeat. Specifically, engaging in sequences of 4–6 Phases (Pooled OR = 1.190; GLMM OR = 1.240) and ≥7 Phases (Pooled OR = 3.265; GLMM OR = 2.593) was associated with an increase in the odds of match defeat compared to short sequences of 1–3 Phases. This suggests that the inability to break the opponent’s defensive line after multiple phases, or the persistence in continuous phase play without achieving tactical advantages, was statistically associated with an adverse outcome within this competitive context. This result aligns with the trends observed by van Rooyen et al. [9], who noted that ruck avoidance was linked to success in the knockout stages of the 2007 Rugby World Cup. Concurrently, this operational pattern contrasts with performance indicators reported in Tier 1 competitions such as the Six Nations [15] or The Rugby Championship [13], where elite winning squads frequently sustain higher structural ball-recycling rates across consecutive phases due to superior continuity skills. In practical terms, our results suggest that prolonging ball possession sequences without establishing strategic advantages may relate to a negative final outcome in the Rugby Europe Championship. From an epidemiological perspective, given that each consecutive phase involves physical contact [34], a game pattern involving ≥7 Phases may entail a greater number of contact opportunities and, within the present sample, was associated with a higher probability of match defeat.

Regarding territorial aspects, the analysis showed an association between pitch location and match outcome. Rucks executed within Own Territory were associated with shifts in outcome probabilities (Pooled OR = 1.481, *p* < 0.001; GLMM OR = 1.492, *p* = 0.001). The estimated marginal means revealed that the absolute predicted probability of a losing outcome was higher when rucks were concentrated in Own Territory (64.8%) compared to Opponent Territory (55.4%). This pattern aligns with traditional rugby union tactics, where failure to clear the ball from the defensive zone increases defensive pressure close to one’s own try line [7,14]. However, from a multivariable perspective, these findings must be interpreted with caution due to potential reverse causation mechanisms, as losing teams frequently accumulate possession and rucks in the opponent’s half during the latter stages of a match to overturn a scoreline deficit [14]. Therefore, future investigations should incorporate time-series or scoreline-stratified analyses to untangle how evolving match status modulates territorial breakdown behaviors. Complementarily, rucks produced Behind Gainline (Pooled OR = 1.478, *p* = 0.001; GLMM OR = 1.663, *p* < 0.001) and On Gainline (Pooled OR = 1.369, *p* = 0.003; GLMM OR = 1.432, *p* = 0.002) were associated with higher odds of defeat compared to rucks where the Gainline was Cleared. These results highlight the tactical importance of the gainline during the ruck for maintaining offensive continuity [35]. From a contextual perspective, prior literature has hypothesized that performing breakdowns while losing forward momentum could alter force transfer mechanisms during impacts; however, since direct biomechanical measurements were not collected in this study, whether this tactical pattern is associated with differences in the physical collision load experienced by players should be examined in future studies using direct biomechanical or wearable-derived measurements [36].

Regarding Player Type and Number of Players in the Ruck, the technical–tactical factors evaluated in the models displayed a significant association with the final match outcome. Rucks involving exclusively Backs (Pooled OR = 1.352) or Backs and Forwards (Pooled OR = 1.077) were contrasted against the Forwards reference baseline. Based on the primary pooled logistic regression model, rucks involving Backs exclusively were associated with the highest estimated marginal probability of defeat among the Player Type categories, reaching 64.4% compared with 57.2% for Forwards. Most notably, the inclusion of the multilevel framework was implemented exclusively as a post hoc sensitivity check to control for the nested dependency of rucks within individual matches. This analysis indicated that the main associations remained broadly consistent after accounting for match-level clustering. Notably, while overpopulating the breakdown with ≥4 Players displayed a non-significant trend in the population-averaged baseline (Pooled OR = 1.297, *p* = 0.068), it became statistically significant in the mixed-effects model (GLMM OR = 1.425, *p* = 0.019) once the within-match dependency was statistically controlled, pointing to an absolute predicted probability of 63.3% compared to the 57.1% baseline for 1–3 Players. Tactically, this pattern might be compatible with slower ball circulation or less effective redistribution of play, although these mechanisms were not directly measured in the present study. From a preventative perspective, this tactical behavior can be discussed alongside prior epidemiological observations in the literature; for instance, longitudinal data in Spanish domestic rugby have shown that forwards sustain a substantial rate of physical trauma due to their constant involvement in close-range contact actions [37]. Furthermore, while Solís Mencía et al. [34] highlighted that grouped play situations generate a high descriptive injury burden within this specific national squad, our data do not monitor medical diagnoses or physical traumas directly. Contesting the breakdown with ≥4 Players could theoretically increase the volume of athletes simultaneously involved in physical contests during ruck skills, combining an inefficient offensive distribution with a higher density of players involved in contact zones [2,34].

Furthermore, the results of this study revealed findings regarding rucks occurring across different periods of the match. Breakdowns occurring during the First Half were associated with lower odds of being observed in matches ending in defeat (OR = 0.625, *p* < 0.001; GLMM OR = 0.607), whereas rucks occurring during the Second Half were associated with a higher estimated marginal probability of defeat within the analyzed sample. This corresponds with an absolute predicted marginal defeat probability of 65.7% during the Second Half compared to 54.5% during the First Half under controlled baselines. This association may reflect tactical or physical deficits accumulated during the first half, or alternatively, a reactive game style imposed by an early scoreline deficit [38,39,40]. Future research incorporating scoreline-stratified analyses would help clarify these mechanisms. From an epidemiological perspective, while the literature on the Spanish national league typically associates higher injury incidence with the third and fourth quarters due to the onset of generalized physical fatigue [37,41], our findings suggest that at the international level, an increased frequency of rucks during the Second Half may combine with cumulative match fatigue, elevating contact exposure and negatively impacting final performance.

### 4.1. Practical Applications

From an applied perspective, the findings of this study provide coaches, performance analysts, and strength and conditioning specialists with contextual benchmarks that could tentatively inform dual performance and injury-prevention frameworks. Rather than offering prescriptive mandates, these data suggest potential considerations for training design. For instance, coaching staff might consider monitoring contact exposure during continuous play, designing attacking sequences that favor rapid ball recycling before sequences become excessively prolonged (e.g., avoiding sequences that exceed 6 game phases), which could theoretically mitigate cumulative physical fatigue from unresolved breakdowns. Concurrently, training tasks could be structured to optimize Number of Players in the Ruck; encouraging rapid ball clearance with only 1–3 players might optimize open-play dynamics while hypothetically reducing the volume of athletes simultaneously exposed to close-range impacts.

### 4.2. Limitations and Future Research

This study is restricted to the observational analysis of 26 matches played by the Spanish men’s senior national team during the Rugby Europe Championship tournament, which limits the immediate generalization of the results to other levels or categories, such as women’s rugby, where distinct collision profiles have already been described [15,18]. Given the observational methodology applied, the model identifies predictive and probabilistic associations rather than direct biomechanical causal relationships, and it lacks simultaneous real-time records of internal fatigue or match injuries. Therefore, future research should integrate contextual ruck exposure variables with kinematic impact measures, workload monitoring, and individual player-specific exposure indicators. This approach is consistent with recent evidence suggesting that multifactorial models combining workload, injury history, fitness, biomechanical, and wearable-derived data may provide more robust injury-prediction frameworks than isolated field-based screening tools [42].

A primary methodological limitation of the present study concerns the statistical independence of observations, given the hierarchical nature of the dataset. The primary logistic regression model was computed at the individual ruck level (*n* = 2575), whereas the dependent variable (match outcome) represents a macro-level construct shared by all rucks executed within a single match (*n* = 26). This nested data structure implies that rucks sharing the same match environment possess a shared variance, which can potentially underestimate standard errors and artificially deflate *p*-values. To mathematically address this within-match dependency constraint, a post hoc multilevel mixed-effects model (GLMM) was implemented as a robustness sensitivity check to explicitly partition within-match covariance from between-match dynamics (τ_00_ = 0.9033; ICC = 0.2154). While the primary pooled logistic regression framework was intentionally maintained in the main text to preserve population-averaged marginal Odds Ratios, which offer direct practical utility for strategic coaching interventions, the comparative parameters presented in Table 1 confirm that the standard errors and tactical indicators remain structurally stable when controlling for the nested match environment. Consequently, these adjusted findings remain broadly consistent with localized probabilistic tactical associations within this competitive framework rather than independent causal chains, mitigating the probability of artificial inflation in the reported significance levels.

Additionally, the distribution of match outcomes in the analyzed sample (1241 victories vs. 1334 defeats) presents a minor variance, which is reflected in the balanced classification performance metrics observed for victories (56.00%) relative to defeats (59.82%). Although this distribution minimizes potential classification bias toward a majority class, the pattern remains consistent with the interpretation offered in the Discussion: defeat-related ruck patterns may be more homogeneous and therefore more consistently captured by the model, whereas victories may depend on a broader and less uniform set of technical, physical, and contextual factors not fully represented among the six modeled variables.

Finally, a methodological limitation concerns model evaluation. Discriminative metrics (AUC, sensitivity, and specificity) were derived directly from the training dataset without internal (e.g., cross-validation or bootstrapping) or external predictive validation. Consequently, these classification statistics reflect strictly in-sample performance. While the post hoc GLMM sensitivity analysis effectively accounted for data clustering across matches, it does not substitute for predictive validation, and the generalizability of these threshold estimates to independent samples remains to be formally tested.

## 5. Conclusions

In conclusion, the present study indicates that technical–tactical ruck behaviors, specifically the accumulated number of phases, positioning relative to the gainline, pitch territory, and the density and type of players involved, display a statistically significant association with international match outcomes. These patterns suggest that lower offensive efficiency during the breakdown is frequently accompanied by a higher probability of match defeat. While these tactical factors explain only a limited share of the overall variability in match outcome, they emphasize the inherently multifactorial nature of performance in elite rugby union. When discussed alongside prior epidemiological concepts, these play patterns in the ruck associated with defeat appear to align with prolonged or high-density contact exposure environments. This suggests that inefficient offensive structures may subject athletes to continuous collision contexts without yielding clear competitive advantages. However, given the observational design and the nested data structure, these relationships should be interpreted as localized probabilistic associations that remained broadly consistent in a post hoc mixed-effects sensitivity analysis, rather than as causal effects or definitive predictive patterns.

## Figures and Tables

**Table 1:** Multivariable Logistic Regression and post hoc Generalized Linear Mixed Model evaluating the statistical associations between technical–tactical ruck indicators and match defeat.

Predictor	Pooled Logistic Model					Generalized Linear Mixed Model				
	*β*	SE	OR	95% CI	*p*	*β*	SE	OR	95% CI	*p*
Intercept	−0.489	0.169	0.613	0.441–0.854	0.004	−0.686	0.26	0.504	0.303–0.838	0.008
Pitch Territory (a)										
Own Territory	0.393	0.094	1.481	1.231–1.781	<0.001	0.4	0.102	1.492	1.221–1.824	0.001
Number of Phases (b)										
4–6 Phases	0.174	0.089	1.19	1.000–1.416	0.05	0.216	0.095	1.241	1.030–1.496	0.024
≥7 Phases	1.183	0.175	3.265	2.316–4.604	<0.001	0.953	0.193	2.593	1.776–3.786	<0.001
Match Half (c)										
First Half	−0.471	0.082	0.625	0.532–0.733	<0.001	−0.5	0.088	0.607	0.511–0.721	<0.001
Player Type (d)										
Backs	0.302	0.112	1.352	1.086–1.684	0.007	0.325	0.123	1.384	1.089–1.760	0.008
Backs & Forwards	0.074	0.104	1.077	0.879–1.320	0.474	0.108	0.112	1.114	0.895–1.388	0.334
Ruck Position (e)										
Behind Gainline	0.391	0.112	1.478	1.187–1.840	0.001	0.508	0.121	1.663	1.312–2.107	<0.001
On Gainline	0.314	0.105	1.369	1.114–1.683	0.003	0.359	0.115	1.432	1.143–1.794	0.002
Number of Players in the Ruck (f)										
≥4 Players	0.26	0.142	1.297	0.981–1.714	0.068	0.354	0.151	1.425	1.060–1.916	0.019

Note: β coefficients calculations derived directly from the full sample pooled framework. Reference categories (OR = 1.000) were: (a) Opponent Territory; (b) 1–3 Phases; (c) Second Half; (d) Forwards; (e) Gainline Cleared; and (f) 1–3 Players. OR: Odds Ratio; CI: Confidence Interval; SE: Standard Error; *p*: *p*-value. Generalized Linear Mixed Model accounts for structural nesting across 26 international matches (*n* = 2575 total rucks) with Match ID entered as a random intercept component (τ_00_ = 0.903, SD = 0.950, ICC = 0.215). Primary pooled model global fit statistics: *χ*^2^ = 137.247, df = 9, *p* < 0.001, Nagelkerke *R*^2^ = 0.069, AUC = 0.621.

## Data Availability

Data and statistical analysis outcomes are hosted in the institutional repository of the University of Murcia (Digitum), available at https://digitum.um.es/entities/publication/10758c7e-4343-46a5-8fed-4cd5587f809b (accessed on 2 June 2026).

## Supplementary Materials

The following supporting information can be downloaded at https://www.mdpi.com/article/10.3390/sports14080333/s1: Supplementary Material S1: STROBE Statement Checklist.

## Abbreviations

The following abbreviations are used in this manuscript:

AUC	Area Under the Curve
CI	Confidence Interval
df	Degrees of freedom
OR	Odds Ratio
P/I/M	Point, idiographic, and multidimensional
ROC	Receiver Operating Characteristic
VIF	Variance Inflation Factor

## References

[1] Fuller C.W., Brooks J.H., Cancea R.J., Hall J., Kemp S.P. (2007). Contact events in rugby union and their propensity to cause injury. Br. J. Sports Med..

[2] Musumeci G., Ravalli S., Amorini A.M., Lazzarino G. (2019). Concussion in sports. J. Funct. Morphol. Kinesiol..

[3] Paul L., Naughton M., Jones B., Davidow D., Patel A., Lambert M., Hendricks S. (2022). Quantifying collision frequency and intensity in rugby union and rugby sevens: A systematic review. Sports Med. Open.

[4] Hendricks S., Till K., den Hollander S., Savage T.N., Roberts S.P., Tierney G., Burger N., Kerr H., Kemp S., Cross M. (2020). Consensus on a video analysis framework of descriptors and definitions by the Rugby Union Video Analysis Consensus group. Br. J. Sports Med..

[5] Sella F.S., McMaster D.T., Serpiello F.R., La Torre A. (2019). Match analysis in Rugby Union: Performance indicators of Rugby Championship and Super Rugby teams. J. Sports Med. Phys. Fitness.

[6] Ungureanu A.N., Brustio P.R., Lupo C. (2021). Technical and tactical effectiveness is related to time-motion performance in elite rugby. J. Sports Med. Phys. Fit..

[7] Guerrero-Esteban S., Solé J., Daza G. (2023). Variables involved in ball possession in rugby: A systematic review. Apunts Educ. Física Deportes.

[8] Bunker R.P., Spencer K. (2022). Performance indicators contributing to success at the group and play-off stages of the 2019 Rugby World Cup. J. Hum. Sport Exerc..

[9] van Rooyen K.M., Diedrick E., Noakes D.T. (2010). Ruck frequency as a predictor of success in the 2007 Rugby World Cup Tournament. Int. J. Perform. Anal. Sport.

[10] Vaz L., Hendricks S., Kraak W. (2019). Statistical review and match analysis of Rugby World Cups finals. J. Hum. Kinet..

[11] Vaz L., Mouchet A., Carreras D., Morente H. (2011). The importance of rugby game-related statistics to discriminate winners and losers at the elite level competitions in close and balanced games. Int. J. Perform. Anal. Sport.

[12] Schoeman R., Coetzee D., Schall R. (2017). Comparisons of performance indicators between Super Rugby and Currie Cup competition during 2014 season. S. Afr. J. Res. Sport Phys. Educ. Recreat..

[13] Vaz L., Carreras D., Mouchet A. (2011). Variables in play which best discriminate between victory and defeat in equally matched rugby matches. Apunts Educ. Física Deportes.

[14] Hendricks S. (2021). Match analysis in rugby. Match Analysis.

[15] Villarejo-García D.H., Navarro-Martínez C., Pino-Ortega J. (2026). Collision volume and contact exposure profile in elite women’s Rugby Union: Differences compared with men. Sports.

[16] Villarejo-García D., Navarro-Martínez C., Pino-Ortega J. (2025). Uso de la estadística multivariante para analizar el rendimiento en competiciones de Futsal: Revisión sistemática. Retos.

[17] Cid F.M. (2016). Estadística Avanzada para Educación Física: Diseño de Experimentos y Estadística Multivariante.

[18] Zheng S., Man X. (2022). An improved logistic regression method for assessing the performance of track and field sports. Comput. Intell. Neurosci..

[19] Thomas J.R., Nelson J.K., Silverman S.J. (2015). Research Methods in Physical Activity.

[20] Anguera M.T., Blanco-Villaseñor A., Hernández-Mendo A., Losada J.L. (2011). Diseños observacionales: Ajuste y aplicación en psicología del deporte. Cuad. Psicol. Deporte.

[21] World Rugby (2023). Laws of the Game Rugby Union.

[22] Soto A., Camerino O., Iglesias X., Anguera M.T., Castañer M. (2022). LINCE PLUS: Research and training in systematic observation of physical activity and sport. Behav. Res. Methods.

[23] American Psychological Association (2017). Ethical Principles of Psychologists and Code of Conduct.

[24] (2016). Regulation (EU) 2016/679 of the European Parliament and of the Council of 27 April 2016 on the Protection of Natural Persons with Regard to the Processing of Personal Data and on the Free Movement of Such Data, and Repealing Directive 95/46/EC. Off. J. Eur. Union.

[25] Villarejo D., Ortega E., Gómez M.-Á., Palao J.-M. (2014). Design, validation, and reliability of an observational instrument for ball possessions in Rugby Union. Int. J. Perform. Anal. Sport.

[26] Losada J.L., Manolov R. (2015). The process of basic training, applied training, maintaining the performance of an observer. Qual. Quant..

[27] Fox J., Weisberg S. (2023). Car: Companion to Applied Regression.

[28] Peduzzi P., Concato J., Kemper E., Holford T.R., Feinstein A.R. (1996). A simulation study of the number of events per variable in logistic regression analysis. J. Clin. Epidemiol..

[29] Riley R.D., Snell K.I.E., Ensor J., Burke D.L., Harrell F.E., Moons K.G.M., Collins G.S. (2019). Minimum sample size for developing a multivariable prediction model: PART II—binary and time-to-event outcomes. Stat. Med..

[30] Colomer C.M.E., Pyne D.B., Mooney M., McKune A., Serpell B.G. (2020). Performance analysis in Rugby Union: A critical systematic review. Sports Med. Open.

[31] Tabachnick B.G., Fidell L.S. (2013). Using Multivariate Statistics.

[32] Hosmer D.W., Lemeshow S., Sturdivant R.X. (2013). Applied Logistic Regression.

[33] Harrell F.E. (2015). Regression Modeling Strategies: With Applications to Linear Models, Logistic and Ordinal Regression, and Survival Analysis.

[34] Solís-Mencía C., Jiménez-Herranz E., Montoya-Miñano J.J., McFall M.F., Aramberri Gutiérrez M., García-Fernández P., Ramos-Álvarez J.J. (2024). Epidemiological study of injuries in the Spanish men’s senior national Rugby XV team. Appl. Sci..

[35] Guerrero-Esteban S., Pardo P., Piedra A., Albesa-Albiol L., Sánchez-Fuentes J.A., Peña-López J., Daza G., Solé J., Caparrós-Pons T. (2023). Variables associated with the performance of a male European professional rugby team: Analysis of the regular season. Retos.

[36] MacLeod S.J., Hagan C., Egaña M., Davis J., Drake D. (2018). The use of microtechnology to monitor collision performance in professional Rugby Union. Int. J. Sports Physiol. Perform..

[37] Murias-Lozano R., Mendía L., Sebastián-Obregón F.J.S., Solís-Mencía C., Hervás-Pérez J.P., Garnacho-Castaño M.V., Maté-Muñoz J.L., García-Fernández P. (2022). The epidemiology of injuries in Spanish Rugby Union División de Honor. Int. J. Environ. Res. Public Health.

[38] Lago C. (2009). The influence of match location, quality of opposition, and match status on possession strategies in professional association football. J. Sports Sci..

[39] Jones N.M.P., Mellalieu S.D., James N. (2004). Performance indicators as a guide to team success in the 2003 Rugby World Cup. J. Sports Sci..

[40] Villarejo-García D.H., Pino-Ortega J., Becerra-Patiño B.A., Navarro-Martínez C. (2026). Performance Determinants in Elite Men’s Sport: A Tactical Analysis of Ball Possession Effectiveness at the 2023 Rugby World Cup. J. Men’s Health.

[41] Murias-Lozano R., San Sebastián-Obregón F.J., Lucio-Mejías H., Saló-Cuenca J.C., Plaza-Manzano G., López-de-Uralde-Villanueva I., Maté-Muñoz J.L., García-Fernández P. (2022). Match injuries in the Spanish Rugby Union División de Honor. Int. J. Environ. Res. Public Health.

[42] Eckart A.C., Ghimire P.S., Stavitz J., Barry S. (2025). Predictive Utility of the Functional Movement Screen and Y-Balance Test: Current Evidence and Future Directions. Sports.

