# Supplementary material for: Analysis of Ruck Contact Exposure and Technical–Tactical Behaviors Related to Match Outcome in International Rugby Union
Source: Sports (Basel). 2026 Aug 3;14(8):333. doi: 10.3390/sports14080333 (PMC13517122; doi:10.3390/sports14080333)
Supplement: Supplementary file 1 [file sports-14-00333-s001.zip › sports-4401003-supplementary.pdf]

STROBE Statement Checklist

Checklist of items that should be included in reports of cross-sectional studies.

Article Title: Ruck Contact Exposure as a Predictor of Match Outcome in International Rugby Union: A Logistic Regression Analysis for Risk Identification

Authors: Diego Hernán Villarejo-García, Carlos Navarro-Martínez, Pedro López Baro, Gabriel Daza-Sobrino, Sebastián Del Rosso, José Pino-Ortega

Section	Item Nº	STROBE Recommendation	Reported Section	Manuscript Context & Specific Details
**Title and Abstract**	**1 (a)**	Indicate the study's design with a commonly used term in the title or the abstract.	**Title / Abstract**	Reported. The abstract explicitly defines the study design: "A retrospective observational study was conducted..."
**Title and Abstract**	**1 (b)**	Provide in the abstract an informative and balanced summary of what was done and what was found.	**Abstract**	Reported. The abstract details the study scope (2,339 rucks from 20 international matches), core predictors, multivariate logit model performance metrics (χ^2 = 144.0, Nagelkerke R^2 = 0.0797, AUC = 0.632, classification accuracy = 58.9%), key Odds Ratios, and main clinical/epidemiological conclusions.
**Introduction**	**2**	Explain the scientific background and rationale for the investigation being reported.	**1. Introduction**	Reported. Paragraphs 1 and 2 justify the work by connecting epidemiological injury loads in elite rugby with open-play performance parameters. Paragraph 3 identifies the lack of direct linkage between match outcome and contextual contact overexposure during rucks.
**Introduction**	**3**	State specific objectives, including any prespecified hypotheses.	**1. Introduction**	Reported. Paragraph 4 formally frames the research question and core hypothesis: "...hypothesized that those ruck variables that prolong exposure... will heighten the probability of defeat..." Paragraph 5 defines the specific study purpose.
**Methods**	**4**	Present key elements of study design early in the paper.	**2.1. Design**	Reported. Early defined as a retrospective, descriptive, and correlational investigation applying a point, idiographic, and multidimensional (P/I/M) observational design.
**Methods**	**5**	Describe the setting, locations, and relevant dates, including periods of recruitment, exposure, follow-up, and data collection.	**2.2. Participants / 2.4. Procedure**	Reported. Matches played by the Spanish senior men's national team during the Rugby Europe Championship in the 2022, 2023, 2024, and 2025 competitive seasons. Audiovisual data hosted by Radio Televisión Española.
**Methods**	**6 (a)**	Give the eligibility criteria, and the sources and methods of selection of participants.	**2.2. Participants**	Reported. Inclusion parameters required official inclusion within the tournament, complete video recording lengths, and sufficient video layout clarity. A total of 2,339 rucks met these technical criteria.

Section	Item Nº	STROBE Recommendation	Reported Section	Manuscript Context & Specific Details
**Methods**	**7**	Clearly define all outcomes, exposures, predictors, potential confounders, and effect modifiers. Give diagnostic criteria, if applicable.	**2.3. Materials and Instruments**	Reported. Explanatory variables are fully defined: Pitch Territory, Number of Phases, Match Half, Player Type, Gainline Progression/Ruck Position, and Number of Players. Dependent variable is match outcome (victory/defeat).
**Methods**	**8***	For each variable of interest, give sources of data and details of methods of assessment (measurement). Describe comparability of assessment methods if there is more than one group.	**2.4. Procedure**	Reported. Logged via a validated multi-dimensional observation tool. Three independent IRB Level 3 certified experts executed the tracking. Inter-observer reliability was robust ($\kappa = .82$) and intra-observer stability was high ($\kappa = .96$).
**Methods**	**9**	Describe any efforts to address potential sources of bias.	**2.4. Procedure / 4.2. Limitations**	Reported. Observational bias minimized through systematic reviewer alignment training and high Kappa verification. Sample size and baseline class distribution bias addressed transparently in section 4.2.
**Methods**	**10**	Explain how the study size was arrived at.	**2.5. Data Analysis**	Reported. Full tournament availability census. Statistical statistical adequacy was confirmed via the events per variable (EPV) calculation, which yielded >185 (928 minority class events / 5 key model predictors), far exceeding the minimum threshold of 10.
**Methods**	**11**	Explain how quantitative variables were handled in the analyses. If applicable, describe which groupings were chosen and why.	**2.3. Materials and Instruments**	Reported. Continuous game situations categorized into logical ordinal metrics following established standards (e.g., phases divided into 1-3, 4-6, ≥7; player density split at ≥4 to capture breakdown overcrowding thresholds).
**Methods**	**12 (a)**	Describe all statistical methods, including those used to control for confounding.	**2.5. Data Analysis**	Reported. Multivariate binary logistic regression was executed to estimate independent regression coefficients (β), standard errors, Z-scores, p-values, and Odds Ratios (OR) with 95% Confidence Intervals while controlling for co-variables.
**Methods**	**12 (b)**	Describe any methods used to examine subgroups and interactions.	**2.5. Data Analysis / 4.2. Limitations**	Reported. Group behavior trends evaluated through Estimated Marginal Means (P(Loss)). Subgroup profiling limitations (gender/competition) detailed as paths for future research.
**Methods**	**12 (c)**	Explain how missing data were addressed.	**2.2. Participants**	Reported. No missing records occurred across the finalized sample because video clarity requirements were enforced as pre-screening inclusion benchmarks.
**Methods**	**12 (d)**	If applicable, describe analytical methods taking account of sampling strategy.	**2.5. Data Analysis / 4.2. Limitations**	Reported. Non-probabilistic whole-population census of available national selection matches. Data nesting effects (rucks within independent matches) are explicitly evaluated as an analytical limitation.
**Methods**	**12 (e)**	Describe any sensitivity analyses.	**2.5. Data Analysis**	Reported. Rigorous checking applied: omnibus sequential likelihood ratio tests, Nagelkerke R^2, Variance Inflation Factors (VIF ranging 1.01-1.14)

Section	Item Nº	STROBE Recommendation	Reported Section	Manuscript Context & Specific Details
				to rule out multicollinearity, sensitivity (0.703), specificity (0.449), and ROC curve (AUC).
**Results**	**13***	Report numbers of individuals at each stage of study—eg numbers potentially eligible, examined for eligibility, confirmed eligible, included in the study, completing follow-up, and analysed.	**2.2. Participants**	Reported. Comprehensive census totaling 2,339 unique rucks tracked across 20 international match fixtures.
**Results**	**14***	Give characteristics of study participants and information on exposures and potential confounders.	**2.2. Participants / 2.3. Instruments**	Reported. Fixed to international male elite rugby union players (Spanish Senior National XV Team). Total exposure context consists of 2,339 rucks across 12 won and 8 lost matches.
**Results**	**15***	Report numbers of outcome events or summary measures.	**2.2. Participants / 2.5. Data Analysis**	Reported. Summary breakdown details 12 victories and 8 defeats, generating 928 ruck instances corresponding to the minority (defeated match) class.
**Results**	**16 (a)**	Give unadjusted estimates and, if applicable, confounder-adjusted estimates and their precision (eg, 95% confidence interval). Make clear which confounders were adjusted for and why they were included.	**3. Results (Table 1)**	Reported. Table 1 outlines fully adjusted multivariate regression metrics simultaneously accounting for all six contextual predictors in the joint logit matrix.
**Results**	**16 (b)**	Report category boundaries when continuous variables were categorized.	**2.3. Materials and Instruments**	Reported. All category boundaries are explicitly detailed: Number of Phases (1–3, 4–6, ≥7) and Number of Players in the Ruck (1–3, ≥4 players).
**Results**	**16 (c)**	If relevant, consider translating estimates of relative risk into absolute risk for a meaningful time period.	**3. Results / 4. Discussion**	Reported. Model logits are extended into absolute risk context through Estimated Marginal Means to isolate exact loss probability shifts (e.g., 66.8% in Own Territory vs. 58.8% in Opponent Territory).
**Results**	**17**	Report other analyses done—eg analyses of subgroups and interactions, and sensitivity analyses.	**2.5. Data Analysis / 3. Results**	Reported. VIF indexes are reported to discard collinearity. Classification margins, classification sensitivity, and specificity thresholds are reported.
**Discussion**	**18**	Summarise key results with reference to study objectives.	**4. Discussion**	Reported. Paragraph 1 recaps core findings showing that tactical ruck combinations match significant outcome trends, noting global fit thresholds (AUC = 0.632; accuracy = 58.9%).
**Discussion**	**19**	Discuss limitations of the study, taking into account sources of potential bias or imprecision. Discuss both direction and magnitude of any potential bias.	**4.2. Limitations and Future Research**	Reported. Extensively discusses potential data clustering/nesting bias (individual rucks nested inside specific matches), class distribution asymmetry, and the absence of an a priori automated cross-validation loop (e.g., LOOCV).
**Discussion**	**20**	Give a cautious overall interpretation of results considering objectives, limitations, multiplicity of analyses, results from similar studies, and other relevant evidence.	**4. Discussion / 5. Conclusions**	Reported. Blends regression data with international performance literature and specialized epidemiological injury burden records (Solís-Mencía, Murias-Lozano), discussing the modest explanatory variance (Nagelkerke R^2 = 0.080).

Section	Item Nº	STROBE Recommendation	Reported Section	Manuscript Context & Specific Details
**Discussion**	**21**	Discuss the generalisability (external validity) of the study results.	**4.2. Limitations and Future Research**	Reported. Discusses external constraints due to observing a single senior tier team within a specific European competition, limiting immediate generalization to women's rugby cohorts.
**Other Info**	**22**	Give the source of funding and the role of the funders for the present study.	**Funding**	Reported. Declared in the final mandatory disclosures section: "This research received no external funding."
